# Investigating the Provision and Context of Use of Hearing Aid Listening Programs From Real-world Data: Observational Study

**DOI:** 10.2196/36671

**Published:** 2022-10-17

**Authors:** Alessandro Pasta, Tiberiu-Ioan Szatmari, Jeppe Høy Christensen, Kasper Juul Jensen, Niels Henrik Pontoppidan, Kang Sun, Jakob Eg Larsen

**Affiliations:** 1 Department of Applied Mathematics and Computer Science Technical University of Denmark Kongens Lyngby Denmark; 2 Department of Applied Mathematics and Computer Science, Cognitive Systems Demant A/S Smørum Denmark; 3 Eriksholm Research Centre Oticon A/S Snekkersten Denmark

**Keywords:** personalized medicine, hearing aids, data logging, listening programs, sound environment, mobile phone

## Abstract

**Background:**

Listening programs enable hearing aid (HA) users to change device settings for specific listening situations and thereby personalize their listening experience. However, investigations into real-world use of such listening programs to support clinical decisions and evaluate the success of HA treatment are lacking.

**Objective:**

We aimed to investigate the provision of listening programs among a large group of in-market HA users and the context in which the programs are typically used.

**Methods:**

First, we analyzed how many and which programs were provided to 32,336 in-market HA users. Second, we explored 332,271 program selections from 1312 selected users to investigate the sound environments in which specific programs were used and whether such environments reflect the listening intent conveyed by the name of the used program. Our analysis was based on real-world longitudinal data logged by smartphone-connected HAs.

**Results:**

In our sample, 57.71% (18,663/32,336) of the HA users had programs for specific listening situations, which is a higher proportion than previously reported, most likely because of the inclusion criteria. On the basis of association rule mining, we identified a primary additional listening program, *Speech in Noise*, which is frequent among users and often provided when other additional programs are also provided. We also identified 2 secondary additional programs (*Comfort* and *Music*), which are frequent among users who get ≥3 programs and usually provided in combination with *Speech in Noise*. In addition, 2 programs (*TV* and *Remote Mic*) were related to the use of external accessories and not found to be associated with other programs. On average, users selected *Speech in Noise*, *Comfort*, and *Music* in louder, noisier, and less-modulated (all *P*<.01) environments compared with the environment in which they selected the default program, *General*. The difference from the sound environment in which they selected *General* was significantly larger in the minutes following program selection than in the minutes preceding it.

**Conclusions:**

This study provides a deeper insight into the provision of listening programs on a large scale and demonstrates that additional listening programs are used as intended and according to the sound environment conveyed by the program name.

## Introduction

### Background

Untreated hearing loss is a widespread condition [1] that has repercussions at an individual [2-4] and societal level [1,5,6]. Globally, over the next 10 years, nearly 1.5 billion people can potentially benefit from having their ear and hearing problems addressed [7]. The adoption of hearing aids (HAs) has been shown to have a positive impact on the quality of life of users [8,9] and mitigate the effect of hearing loss on household income [4]. However, one of the requisites for the widespread adoption and use of HAs is user satisfaction [10]. HA users use HAs and report listening difficulties in different real-life situations, ranging from face-to-face conversations to coping with environmental sounds [11]. Therefore, to achieve high user satisfaction, HAs need to be able to cater to a wide range of situations. This is confirmed by previous research that found that one of the main reasons for not owning or not using HAs is that they do not work well in specific situations, for instance, when there is background noise [10,12,13], when listening to speech [14], or when being in a large group of people [15]. HA users can benefit from certain HA features in specific listening environments [16]. For instance, noise reduction has been found to improve noise tolerance [17] and to decrease sustained listening effort in low signal-to-noise ratio environments [18], although its impact on speech intelligibility is equivocal [17,19,20].

Therefore, programmable multimemory HAs have been introduced, which enable providing the user with multiple listening programs for specific listening situations. Currently, 41% of HA owners have such programs [21]. Listening programs set predefined rules for contextually adapting different audiological parameters such as overall gain, frequency shaping of the gain, noise reduction, and directionality. Programs can be manually selected via the HA buttons, a remote control, or a smartphone app. Users are usually advised to use a program in a specific listening situation [22]. This is reflected by the name of the program, which often conveys the situation where it is meant to be used (eg, *Speech in Noise* and *Music*) [22]. Thus, programs are a way for users to contextually adapt the device settings in specific listening situations and thereby personalize their listening experience. Therefore, investigating the use of listening programs potentially enables a deeper understanding of users’ behavior and needs.

### Related Work

To benefit from listening programs, HA users need to be able to characterize the listening environment adequately and actively select the appropriate program [22]. Previous research conducted on 11 experienced HA users has shown that the percentage of users who selected identical programs in the same situation (repeatability) surpassed the level corresponding to pure guess under almost all listening conditions [23]. Higher repeatability has been found in demanding listening situations [23]. These results suggest that listening programs can discernibly impact the listening experience.

Although different listening programs can potentially be beneficial and discernible for HA users, little is known about their real-world use. De Graaff et al [22] performed a scoping review on the use of multimemory devices containing several listening programs and investigated whether HA users appreciate and adequately use the option to switch between programs. Remarkably few studies were found on the use of multiple programs for various listening environments. Stelmachowicz et al [24] found that HA users did not tend to select different settings (in terms of frequency shaping of the gain) across simulated sound environments, although differences in the preferred overall gain were sometimes observed. Conversely, Keidser et al [25] found that 5 out of 27 HA users preferred different frequency response characteristics in different listening conditions, mainly in noisy environments. Similarly, Banerjee [26] found that HA users preferred the default setting most often and nondefault settings mainly in difficult listening situations. In addition, several studies found that most HA users switched between omnidirectional and directional microphone settings and that microphone preferences depend on the characteristics of the listening environment [27-30].

These studies suggest that some HA users value and use the option to switch between listening programs. However, the existing literature is sparse and dated.

While listening programs investigated in older studies used to set a constant level for an audiological parameter (eg, higher constant amount of noise reduction), nowadays listening programs set dynamic rules for contextually adapting the parameters (eg, rules that provide earlier and stronger noise reduction as the user transitions to a complex environment). However, some questions remain unanswered. First, it is not clear what motivates an HA user to obtain a multimemory HA and manually switch between programs and in which listening situations users particularly seek device personalization. Second, as highlighted in the aforementioned systematic review, little is known about the correct use of programs designated for a specific listening environment [22]. Indeed, establishing the need for a multimemory device does not guarantee that the user will immediately notice the benefits of multiple programs. The failure to match the multimemory HA settings to the communication and environmental needs of the individual may lead to delays in fully realizing its benefits [31]. None of the studies included in the systematic review examined whether a certain program was used in the correct listening environment (eg, whether users selected a *Speech in Noise* program in noisy environments) during everyday life [22].

Furthermore, most of these studies relied on self-reported measures collected over a short period. Indeed, they used diaries or questionnaires in which HA users reported use, preferences, and details of the listening environments. Whether the appropriate program is used in each listening environment cannot be derived from these data [22]. Moreover, most studies have paid little attention to the continuation of use of the listening programs after the completion of the study. On the one hand, participants might use programs during the study period but stop using them once the study finishes. On the other hand, they might need to acclimatize to the use of programs, and their preferences may only be evident after extended use [32]. In contrast to self-reported measures, data logging enables investigating the real-world behavior of a larger number of users [33]. It allows gathering objective data about program use and objective contextual data. Moreover, it enables assessing program use with a greater temporal resolution and longitudinally making it possible to investigate detailed patterns of use, explore the long-term user behavior, and account for the acclimatization phase [34]. Investigating the use of listening programs by using objective data logging could unveil insights into how users select different listening programs under natural conditions, thereby paving the way for more personalized hearing care solutions.

### Research Objective

We aimed to investigate the provision and context of use of multimemory HAs by leveraging objective data logged by smartphone-connected HAs from in-market users across several countries. First, we investigated the provision of multiple listening programs for various listening environments. Namely, we examined how many and which programs HA users have and use and whether some programs are commonly provided together. Second, we explored whether HA users use specific programs in distinct listening situations and whether such situations reflect the listening intent conveyed by the name of the program. We did so by focusing on users who repeatedly use specific programs and investigating the sound environment in which such programs are selected.

## Methods

### Participants and Apparatus

This study used data from a large-scale internal (Oticon A/S) database, which stores logs of HA use of HA owners who have signed up for the HearingFitness feature [35] via the Oticon ON smartphone app. The participants were the owners of Oticon Opn HAs who used the HearingFitness feature between June and September 2020. In the sign-up process, the participants actively gave their consent for data to be collected, stored, and used for research purposes on aggregated levels. No personal identifiers were collected.

### Ethics Approval

No additional ethics approval was necessary for this study according to the Danish National Scientific Ethical Committee [36].

### Data and Data Analysis

Using the fitting software, the hearing care professional can provide an HA user with up to 4 listening programs (ie, one for each of the 4 memory slots available in the HAs), by selecting from a list of predefined listening programs, by fine-tuning and renaming predefined listening programs, or by freely creating new ones. The hearing care professional can decide on both the quantity and the order of the provided listening programs by assigning a specific program to the preferred memory slot. In addition, when the user uses some accessories (eg, television adapter and remote microphone), the HA adds special programs on top of the 4 available memory slots.

When the HAs are connected to the smartphone, the HearingFitness feature logs time-stamped data about the interactions with the HAs, such as the selection of specific listening programs. To account for different phrasing or different languages adopted by hearing care professionals when naming the programs, similar program names were coded in fewer categories. Moreover, when the HAs were connected to the smartphone, time-stamped continuous data about the sound environment were collected every 10 minutes, and every time a listening program was selected by the user. Such data represent acoustic characteristics of the momentary sound waves sensed by calibrated HA microphones at ear level. Namely, the sound pressure level (SPL), the noise floor (NF), and the sound modulation level (SML) in decibels were measured across a broad frequency band (0.1-10 kHz) [37].

The SPL is the level output estimate from a low-pass infinite impulse response filter with a time constant of 63 milliseconds [38]. The SPL is the most used indicator of the sound wave strength and correlates well with the human perception of loudness [39]. A bottom tracker (peak detector) of the SPL is implemented with a slow dynamic attack time of 1 to 5 seconds and a fast release time of 30 milliseconds. A top tracker (valley detector) is implemented with the reverse [38]. The NF is the level of background noise in a signal and is estimated based on the bottom tracker of the SPL. The SML is derived as the difference between a top and bottom tracker of the SPL [38]. The SML describes how much the modulated variable (eg, speech) of the signal varies around its unmodulated level and can be viewed as an estimator of the temporal signal-to-noise ratio without having to separate the signal and noise.

### Provision of Listening Programs

The provision of listening programs was investigated by including users who have usage information for at least 20 hours and analyzing, for each user, the programs that have been selected at least once in the 4-month period. The 20-hour threshold was adopted to ensure that the program provision was evaluated for users logging sufficient data while still including as many users as possible.

We explored the provision of listening programs by computing the number of programs provided per user and by analyzing the name and usage of the most frequently provided programs. Furthermore, we investigated the relationships between programs by determining the association rules [40] using the Apriori algorithm [41]. Such an algorithm enables exploring how 2 or more listening programs are related to one another by analyzing the programs that are frequently provided together. Given a set of n programs P={*p_1,_p_2_,…,p_n_}* and a set of users U={*u_1_,u_2_,…,u_m_}*, where each user is provided with a subset of the programs in P, a rule is defined as an implication of the form X⇒Y, where X is the antecedent, Y is the consequent, X,Y⊆P, and X∩Y=∅ [41]. In determining the association rules, the default program (ie, *General*) was excluded. Indeed, the default program is available (chosen or prescribed) for nearly all users and including it in the association rules would not be of interest. Instead, the association rules related to the 5 most frequent additional listening programs were inspected. The rules were evaluated based on several metrics, including support, coverage, confidence, and lift [41]. The support of a rule defines how often the rule appears in the data set. The coverage refers to how often the antecedent of a rule appears in the data set and measures how often the rule can be applied [42]. The confidence of a rule is defined as conf(X⇒Y)=support(X∪Y)/support(X) and can be interpreted as an estimate of the probability P(Y|X) [41], measuring how often a rule is correct out of the applicable cases. A potential issue with confidence is that an association rule having a very frequent consequent will always have high confidence. The lift addresses this concern by considering how frequent the items are in the data set. The lift of a rule is defined as lift(X⇒Y)=support(X∪Y)/(support(X)support(Y)) and can be interpreted as the deviation of the support of the whole rule from the support expected if the antecedent and the consequent were independent [41]. Finally, the likelihood of a program being provided to users with 1, 2, 3, or 4 programs was investigated. The data manipulation was performed in Python (Python Software Foundation). The association rule mining was performed in R (R Foundation for Statistical Computing) by using the *arules* package [43].

### Use of Listening Programs Versus Sound Environment

Contextual program use was evaluated by analyzing the sound environment (SPL, NF, and SML) during program selection. For each logged selection of a specific listening program, the sound environment measured in a 10-minute time window centered on the program selection was considered (ie, 5 minutes preceding and 5 minutes following the selection). For each program, only users with at least 5 selections were included. Such a threshold was chosen to ensure that users’ behavior was inferred from a representative sample of program selections while, at the same time, not discarding too many users. Moreover, based on the analysis described in the *Provision of Listening Programs* section, only a relevant subset of the listening programs was included.

For visualization purposes, the sound environments occurring during repeated selections of a specific program by the same user were averaged. We visually compared the distribution of users by their average sound environments occurring when selecting a specific listening program versus their average sound environments occurring when selecting the default program (ie, *General*).

Owing to the unbalanced nature of the data (unequal samples per participant, hour, etc), associations between program selections and sound environment were analyzed by using linear mixed effect (LME) models, as recommended by Oleson et al [44]. Specifically, SPL, NF, and SML were treated as dependent variables in 3 separate random intercept models defined as the following:


*Y_ijk_ = β_0_+β_1_PROGRAM_ijk_+u_0j_+v_0k_+e_ijk_,
*

*i=1,…,I, j=1,…,J,k=1,…,K*
**(1)**

where *i* indexes all observations (I=332,271 program selections), *j* indexes the participants (J=1312), *k* indexes the time of the day (K=24), and *Y* is the sound environment (average SPL, NF, and SML in 3 separate models) occurred in a 10-minute time window centered on program selection. The selected listening program (*PROGRAM*) was treated as fixed effect, while 
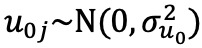
 and 
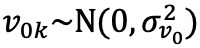
 are the random intercepts, respectively for the *j*-th participant and *k*-th time of day (in hours).

In addition, to account for differences in participant behavior, we fitted the data with 3 random intercept and slope models defined as the following:


*Y_ijk_=β_0_+β_1_PROGRAM_ijk_+u_0j_+v_0k_+u_1j_PROGRAM_ijk_+e_ijk_, i=1,…,I, j=1,…,J,k=1,…,K*
**(2)**

where compared with the simpler model (equation 1), the only additional term is *u_1j_PROGRAM_ijk_*, where 
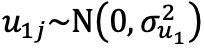
 is the random slope varying across participants for the program effect. These relatively more complex models (equation 2) were compared with simpler models (equation 1) by conducting likelihood ratio tests.

Furthermore, we investigated whether the sound environment changed before or after the program selection by analyzing the sound environment measured in the 5 minutes preceding each program selection and the 5 minutes following it. The difference in sound environment before and after program selection was assessed by 3 separate LME models defined as the following:


*Y_ijk_=β_0_+β_1_PROGRAM_ijk_+β_2_TIMEWINDOW_ijk_ +β_3_PROGRAM_ijk_×TIMEWINDOW_ijk_+u_0j_+v_0k_+e_ijk_, i=1,…,I, j=1,…,J,k=1,…,K*
**(3)**

where *i* indexes all observations (I=273,687 program selections), *j* indexes the participants (J=825), *k* indexes the time of the day (K=24), and *Y* is the sound environment (average SPL, NF, and SML in 3 separate models). The selected listening program (*PROGRAM*) and the time window (*TIMEWINDOW*, ie, *5 minutes before* or *5 minutes after*) were treated as fixed effect. The interaction between *PROGRAM* and *TIMEWINDOW* was introduced to test whether the difference in sound environment levels before and after program selection depends on which program is selected. Finally, 
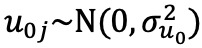
 and 
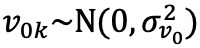
 are the random intercepts, respectively, for the *j*-th participant and *k*-th time of day (in hours). By conducting likelihood ratio tests, these models were compared with simpler models excluding *PROGRAM*. Moreover, by conducting post hoc ANOVA tests, the significance of the variables included in equation 3 was tested. Finally, pairwise comparison (ANOVA) tests were performed on the estimated marginal means from the interaction model to test the difference in the sound environment before and after selection of each listening program.

The data manipulation and visualization were performed in Python using the NumPy [45], Pandas [46], Seaborn [47], and Scipy [48] libraries. The data analysis was performed in R using base functions, and the *lmerTest* (version 3.13 [49]) and *emmeans* (version 1.74-1 [50]) packages were used to apply LME modeling.

## Results

### Provision of Listening Programs

The data processing described in the *Methods* section resulted in a total of 32,336 users and 67,996 programs provided. On average, the sampled users had a connected HA use of 5.88 hours per day. However, when only considering days with at least 1 hour of connected HA use, the average connected HA use amounted to 8.81 hours per day.

**Figure 1:**
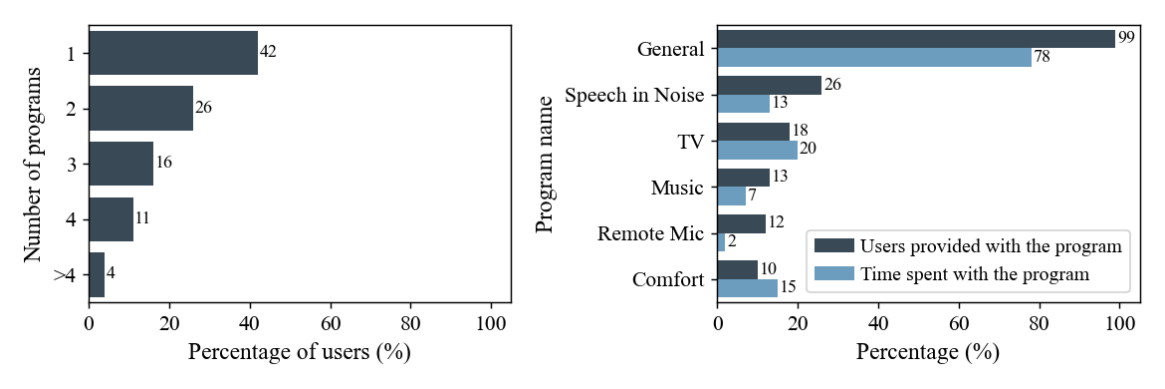
Left, the number of listening programs available for each user is displayed. Right, the provision and usage of the 6 most frequently provided programs are presented. The percentage of users provided with each of the 6 programs (dark blue bars) and the percentage of usage time spent with the programs (light blue bars) are shown. The percentage of usage time spent with each program is computed for the users having that program and at least another program.

Among the HA users, 57.71% (18,663/32,336) had >1 listening program (Figure 1). Almost every user (31,871/32,336, 98.56%) had the default program, *General* (Figure 1). This means that more than half of the users have at least one program for specific listening situations in addition to the default program. Furthermore, 25.8% (8344/32,336), 12.98% (4199/32,336), and 10.26% (3319/32,336) of the users had a *Speech in Noise*, *Music*, and *Comfort* program, respectively. The names of these programs convey a specific listening intent. In addition, 18.13% (5862/32,336) and 11.67% (3773/32,336) of the users had a *TV* and *Remote Mic* program, respectively. These programs are related to the use of an accessory, such as a television adapter and a remote microphone.

In addition to the provision of programs, their use was investigated by computing the percentage of time spent in each program for users with that program and at least another program. *General* was the most used program, accounting on average for 78% of the HA use time. *Speech in Noise*, *Music*, and *Comfort*, respectively, accounted for 13%, 7%, and 15% of HA use time. *TV* and *Remote Mic* accounted for 20% and 2% of HA use time, respectively.

**Figure 2:**
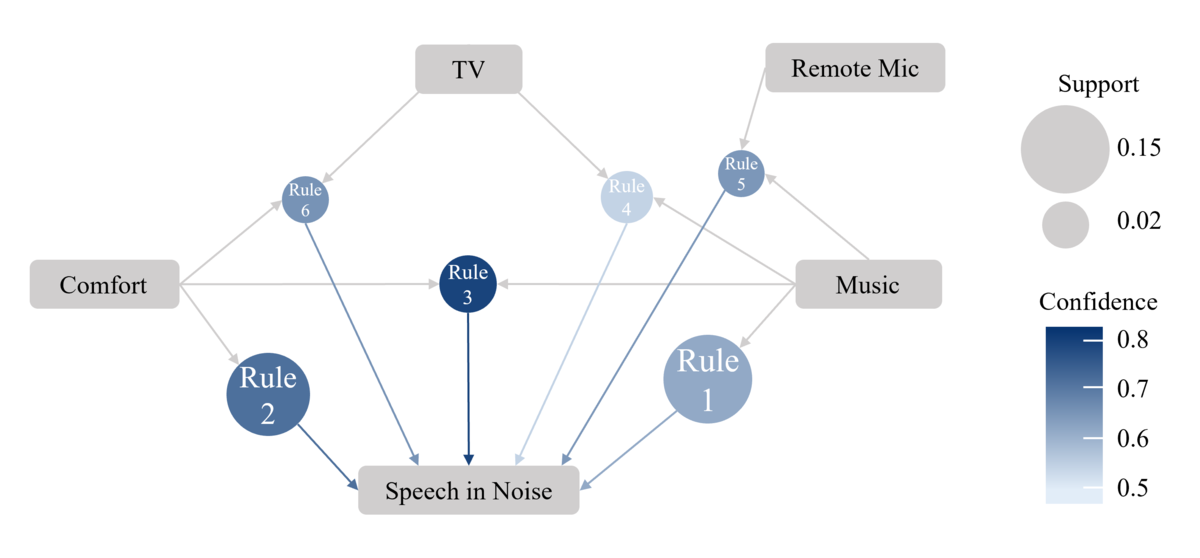
Association rules with support ≥0.02, confidence >0.5, and lift >1 (see the Provision of Listening Programs section). The support of each rule is indicated by the area of the circle, while the confidence is conveyed by the color intensity. *Speech in Noise* is the consequent of all rules, suggesting that it is a primary program, frequently provided when secondary programs such as *Comfort* and *Music* are also provided.

Investigating the association rules with support ≥0.02 and confidence >0.5 (Figure 2) enables exploring the relationships between programs. In this analysis, *General* was not considered as it is uniformly provided and is not an additional listening program. The detailed metrics of the selected rules are presented in Table 1. *Speech in Noise* was not only the most common additional listening program but also a primary program that users get when also getting secondary programs. Indeed, *Speech in Noise* was the consequent of all selected rules, while either *Comfort* or *Music* was always in the antecedent set. As shown by the confidence metric in Table 1, 62.2% (2612/4199) and 71.01% (2357/3319) of the users who had either *Music* (rule 1) or *Comfort* (rule 2), respectively, also had *Speech in Noise*. Similarly, 78.76% (801/1017) of the users who had both *Music* and *Comfort* (rule 3) also had *Speech in Noise*. For these rules, the lift is >1, indicating that users are more likely to have *Speech in Noise* when they also have *Music* or *Comfort*. In contrast, although *TV* was a frequently provided program, users who had such programs were not more likely to have other listening programs.

**Figure 3:**
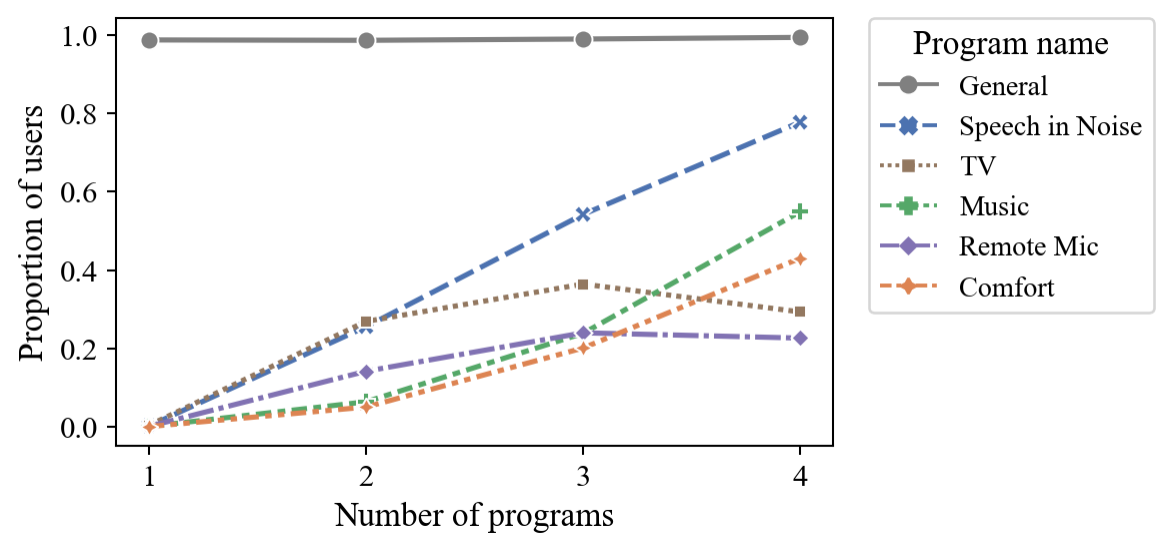
Likelihood of specific listening programs being provided to users with 1, 2, 3, or 4 programs.

Figure 3 confirms some of the previous findings. Almost all users have the *General* program regardless of the number of additional programs. Among the users that have 2 programs, *Speech in Noise*, *TV*, and, to a lesser extent, *Remote Mic* are more likely to be available than *Music* and *Comfort*. For users with 3 or 4 programs, the likelihood of having the primary program *Speech in Noise* grows linearly, the likelihood of having *TV* or *Remote Mic* remains relatively constant, and the likelihood of having secondary programs *Music* and *Comfort* increases.

**Table 1:** Association rules with support ≥0.02, confidence >0.5, and lift >1.

Rule	Antecedent	Consequent	Support	Coverage	Confidence	Lift	Count
1	Music	Speech in Noise	0.14	0.23	0.62	1.35	2612
2	Comfort	Speech in Noise	0.13	0.18	0.71	1.54	2357
3	Comfort and Music	Speech in Noise	0.04	0.06	0.79	1.71	801
4	Music and TV^a^	Speech in Noise	0.03	0.05	0.56	1.21	476
5	Music and Remote Mic	Speech in Noise	0.02	0.03	0.66	1.43	401
6	Comfort and TV	Speech in Noise	0.02	0.03	0.67	1.45	377

^a^TV: television.

### Use of Listening Programs Versus Sound Environment

Since the findings presented in the *Provision of Listening Programs* section, we investigated the sound environments in which a relevant subset of the listening programs was used. We focused on programs that convey a specific listening intent, whether they are primary (*Speech in Noise*) or secondary (*Comfort* and *Music*). These 3 programs are meant to be used in specific listening situations and are not related to the use of an accessory. The data processing described in the *Methods* section resulted in a total of 332,271 program selections from 1312 users.

**Figure 4:**
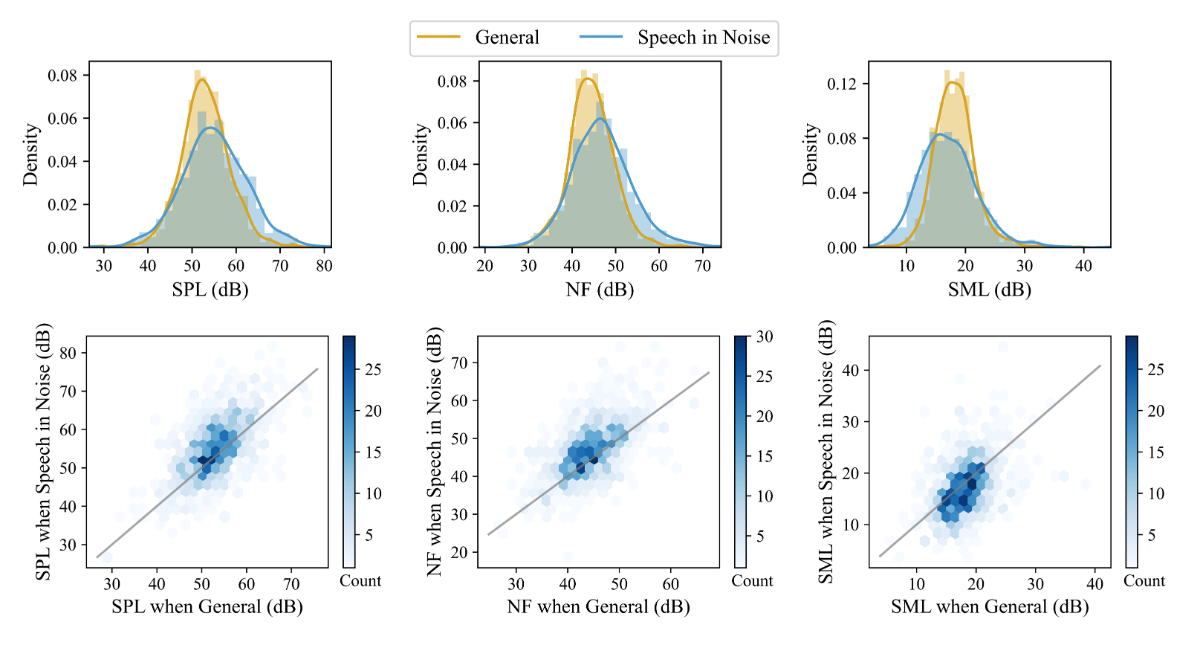
Analysis of the sound environment (sound pressure level [SPL], noise floor [NF], and sound modulation level [SML]) in which *Speech in Noise* and *General* are selected. Compared with *General*, users select *Speech in Noise* in louder, noisier, and less-modulated environments. In the upper figures, distribution of users (using histograms and kernel density estimation) by their average sound environment when selecting *General* and *Speech in Noise*. In the lower figures, 2D histograms displaying, for each user, the sound environment when selecting *Speech in Noise* (y-axis) and *General* (x-axis). The color of the hexagon is determined by the number of users in the hexagon. The identity line (y=x) is drawn in gray. If a user experiences the same sound environment when selecting *Speech in Noise* and *General*, the corresponding hexagon falls exactly on the identity line.

**Figure 5:**
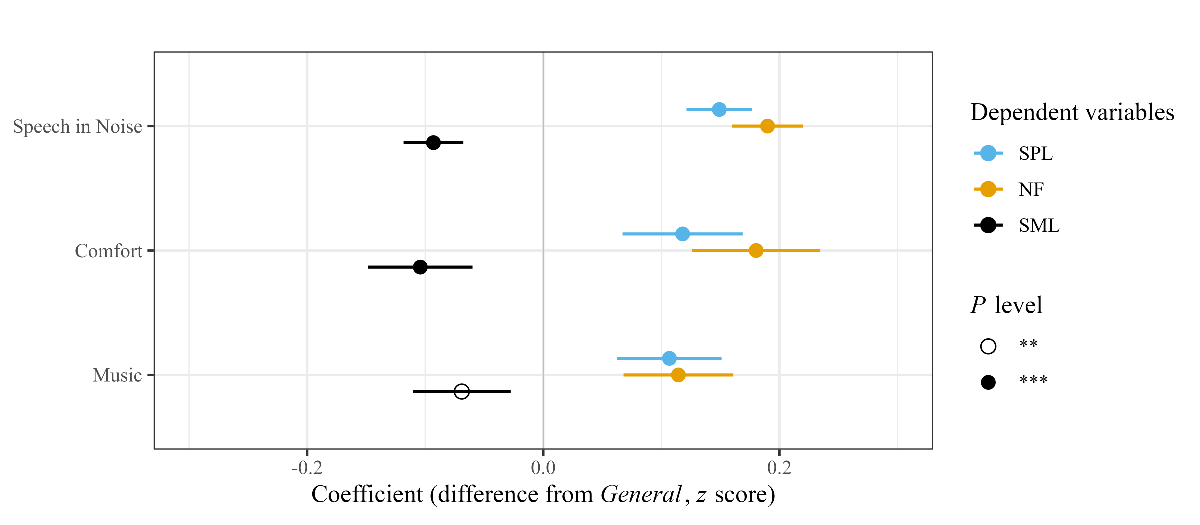
Coefficients and 95% CIs for predicting sound pressure level (SPL), noise floor (NF), and sound modulation level (SML) based on the selected listening program (random intercept and slope model). The baseline condition is the *General* program, so the coefficients quantify the difference in standard score between the sound environment when selecting *Speech in Noise*, *Comfort*, or *Music*, and the sound environment when selecting *General*, computed in a 10-minute interval centered on the program selection. Note that 3 separate models were fitted for predicting the 3 sound environment variables (SPL, NF, and SML). **P<.01; ***P<.001.

First, we analyzed whether the primary program (*Speech in Noise*) was selected in different listening situations compared with the default program (*General*). The upper graphs in Figure 4 display the distribution of users by their average sound environment respectively when selecting *Speech in Noise* and *General*. Users selected *Speech in Noise* in louder (higher SPL), noisier (higher NF), and less-modulated (lower SML) sound environments. Indeed, on average, users selected *Speech in Noise* at 55.1 (SD 7.4) dB SPL, 46.9 (SD 7.0) dB NF, and 17.1 (SD 4.9) dB SML, while they selected *General* at 53.0 (SD 5.6) dB SPL, 44.5 (SD 5.2) dB NF, and 18.2 (SD 3.5) dB SML. The likelihood ratio tests documented that the more complex LME models (equation 2, ie, random intercept and slope for each participant) fit the data significantly better than the simpler model (equation 1) with only a random intercept for each participant (SPL: *χ^2^_10_=3103.7, *P*<.001*; NF: *χ^2^_10_=4308.7, *P*<.001*; MI: *χ^2^_10_=1806.6, *P*<.001*). The more complex model (equation 2) was fitted by setting the *General* program as the baseline condition. The coefficients of the more complex LME models (Figure 5) confirmed that *Speech in Noise* and *General* were selected in different sound environments in terms of SPL, NF, and SML (all *P*<.001). The coefficients also indicate that the scale of the difference ranges from around 0.09 to 0.19 SDs (z-score); that is, 9% to 19% of the overall SD. Moreover, inspecting individual users, the lower graphs in Figure 4 corroborate the LME outcomes and show that most of the users (614/963, 64%; 633/963, 66%; and 593/963, 62%, respectively) selected *Speech in Noise* in environments characterized by higher SPL, higher NF, and lower SML.

Second, we analyzed whether the secondary programs (*Comfort* and *Music*) were selected in specific listening situations. As shown in Figure 5, users selected both the programs in louder, noisier, and less-modulated (coefficients of LME models, all *P*<.01) sound environments compared with the sound environment in which they selected *General*. Subsequently, equation 2 was refitted by changing the contrast so that the *Speech in Noise* program represented the baseline condition. This made it possible to compare whether *Comfort* and *Music* were selected in different listening situations compared with *Speech in Noise*. *Comfort* was selected in less-loud (β=–0.029, SE 0.012, *P*=.014) and less-modulated (β=–0.031, SE 0.0125, *P*=.013) environments, whereas *Music* was selected in less-loud (β=–0.083, SE 0.011, *P*<.001) and less-noisy (β=–0.086, SE 0.0103, *P*<.001) environments.

**Figure 6:**
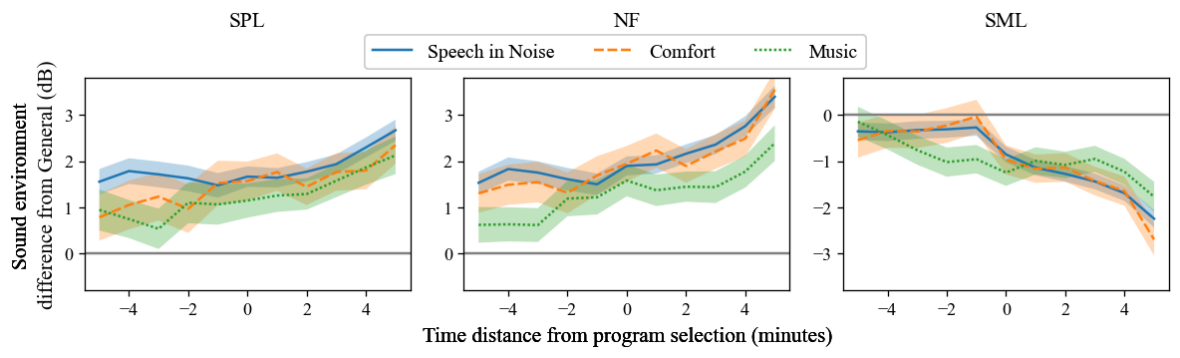
The 5-minute running average (SE) of the sound environment difference from *General*, computed in a time window near the program selection (ie, the solid gray line represents the sound environment when the *General* program was selected). The difference deviates from 0 throughout the whole time window. However, especially for NF and SML, the difference increases after program selection. SPL: sound pressure level; NF: noise floor; SML: sound modulation level.

Finally, we investigated the extent to which the sound environment changed from before to after the program selection. Figure 6 shows, for a time window near the program selection, the 5-minute running average of the difference between the sound environment when selecting a program and when selecting *General*. For all 3 programs (*Speech in Noise*, *Comfort*, and *Music*) and all 3 sound environment features (SPL, NF, and SML), a difference from *General* was observed throughout the whole 10-minute time window. In addition, the sound environment difference appeared to increase after program selection.

The likelihood ratio tests (SPL: *χ^2^_6_=711.0, *P*<.001*; NF: *χ^2^_6_=1597.4, *P*<.001*; and SML: *χ^2^_6_=749.2, *P*<.001*) showed that the more complex models (equation 3, including *PROGRAM*, *TIMEWINDOW*, and *PROGRAM×TIME WINDOW*) fit the data significantly better than the simpler model (only including *TIMEWINDOW*).

**Figure 7:**
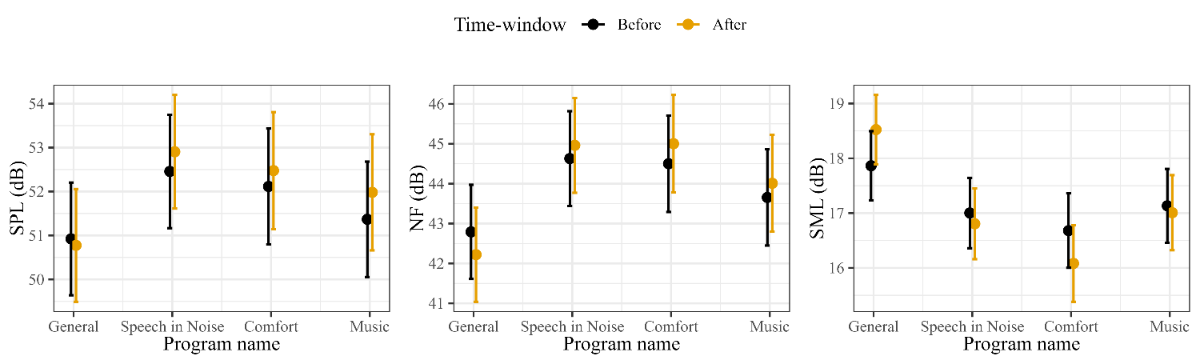
Predicted values of sound pressure level (SPL), noise floor (NF), and sound modulation level (SML) by selected *PROGRAM* (ie, *General*, *Speech in Noise*, *Comfort*, and *Music*) and *TIMEWINDOW* (*before*, ie, the 5-minute time window before program selection; *after*, ie, the 5-minute time window after program selection). Error bars represent the 95% CIs.

Moreover, post hoc ANOVA tests revealed that the interaction between *PROGRAM* and *TIMEWINDOW* was significant for all 3 sound environment features (all *P*<.001). This suggests that the difference in sound environment before and after program selection depends on the specific program. The marginal effects predicted by the interaction term *PROGRAM×TIMEWINDOW* are shown in Figure 7. Pairwise comparisons (*Before* and *After*) confirmed that the sound environment gets quieter, less noisy, and more modulated (all *P*<.01) in the time window after the selection of *General* (compared with the time window before the selection). In contrast, the sound environment became louder, noisier, and less modulated (all *P*<.05) after the selection of *Speech in Noise* (compared with before the selection); noisier and less modulated (both *P*<.001) after the selection of *Comfort*; and louder and noisier (both *P*<.05) after the selection of *Music*.

## Discussion

### Principal Findings

This study investigated the provision and context of use of HA listening programs by analyzing real-world data logged through smartphone-connected HAs.

Most HA users in our sample (18,663/32,336, 57.71%) were found to have listening programs for specific listening situations in addition to the *General* default program. According to a previous study analyzing self-reported data, 41% of HA owners have a program button or switch to change the HA response for different listening environments [21]. The inclusion criteria (ie, users of the HearingFitness feature via a smartphone app) and the data collection method (ie, objective data logging) of our study could explain the higher prevalence of listening programs. Among users having access to the default program and to at least one additional program, the default program was used 78% of the time. This is consistent with a previous study that estimated the default setting to be suitable 75%-85% of the time [26].

In addition to the default program, *Speech in Noise* was the most commonly provided program. By association rule mining, *Speech in Noise* was also found to be a primary additional program that users tend to get when also getting other secondary programs, such as *Comfort* and *Music*; that is, it rarely occurs that users are provided with *Comfort* and *Music* but not with *Speech in Noise*. This suggests that when users either request or are recommended additional listening programs for specific listening situations, *Speech in Noise* is provided as the primary step. This finding is consistent with previous studies reporting that HA users most frequently struggle when there is background noise [10,12,13] or when they are in a large group of people [15], and consequently, they are least likely to be satisfied with their hearing when following conversations in noise and in large groups [21]. *Comfort* and *Music* resulted to be secondary programs, frequently provided in combination with *Speech in Noise* and more likely to be provided to users having 3 or 4 programs. Similar to *Speech in Noise*, these programs signal the interest in personalizing the listening experience in a specific listening situation; that is, when it is noisy but there is no need to communicate and when listening to music. Although these situations are not as prevalent as communicating in noise, users highly motivated to personalize their experience can still benefit from adopting specific listening programs for these situations. The prevalence of the *Music* program is consistent with previous studies finding that between 30% and 67% of HA users may encounter difficulties with listening to music [51,52] and indicating that the enjoyment of listening to music with HAs could be improved by addressing problems such as distortion, acoustic feedback, insufficient or excessive gain, unbalanced frequency response, and reduced tone quality [52,53]. A listening program dedicated to music has previously been proposed to make music more enjoyable [54].

Despite being common programs, *TV* and *Remote Mic* were provided differently than the other programs. They were frequently provided to users having only 2 programs (including *General*), but they were not frequently provided in connection with other additional programs, and their prevalence did not increase among users having >2 programs. This might be explained by the fact that such programs are related to the use of a television adapter (ie, a device that enables streaming the television sound to the HAs) or a remote microphone. Therefore, such programs show an interest in using the accessory more than in contextually adapting the HA settings through a listening program. The *TV* program was the most used program (20% of the time) besides the default program. In contrast, the *Remote Mic* program was only used 2% of the time. These findings suggest that the television adapter is extensively used by its owners, while the remote microphone is used in isolated occasions. In addition, it should be noted that selecting the *TV* program can actively modify the sound environment by either silencing the television or maintaining a normal level for other members of the household while reproducing the sound directly into the HAs. Therefore, *TV* and *Remote Mic* were not included in the sound environment analysis.

Subsequently, we analyzed the sound environment in which *Speech in Noise*, *Comfort*, and *Music* were selected. First, we found that, on average, users selected *Speech in Noise* in louder, noisier, and less-modulated environments compared with the environment in which they selected *General*. This proves that HA users select the *Speech in Noise* program in environments that possess distinct characteristics and that better resembles a conversation in noise. Second, *Comfort* was also selected in louder, noisier, and less-modulated listening environments compared with *General*, suggesting that HA users select it when they want to get relief in noisy environments. Interestingly, HA users selected *Comfort* in less-loud and less-modulated environments than when selecting *Speech in Noise*, indicating that *Comfort* is activated in situations with fewer auditory signals and likely with the intent of increasing the pleasantness of nonspecific listening. Third, *Music* was selected in louder, noisier, and less-modulated listening environments compared with *General*, but in less-loud and less-noisy environments compared with *Speech in Noise*. The music playing in the environment might explain the higher loudness and noise, although not as extreme as the *Speech in Noise* scenarios. Overall, considering that HA users are typically counseled to use a program in a specific listening situation [22], our findings suggest that they tend to follow such recommendations in the real-world use of their HAs. Moreover, the random intercept and slope model (equation 2) significantly outperformed the intercept model (equation 1), suggesting that the effect of program selection on sound environment varies among participants. Empowering users to personalize their listening experience by contextually adapting the HA settings can therefore result in more appropriate settings for some relevant listening situations.

Finally, we analyzed how the sound environment changes from a time window preceding a program selection to a time window following the program selection. For all 3 acoustic predictors, the sound environment change was different when selecting *Speech in Noise*, *Comfort*, or *Music* than when selecting *General*. Specifically, the sound environment gets louder, noisier, and less-modulated in the time window following a selection of *Speech in Noise*, while a selection of *Comfort* leads to nosier and less-modulated environments, and a selection of *Music* leads to louder and nosier environments (Figure 7).This suggests that some users tend to select additional listening programs in anticipation rather than as a reaction, to a more complex sound environment, and that the acoustic features can discriminate between them. In contrast, the sound environment gets quieter and more modulated after the selection of *General*. This indicates that some users tend to select the default program in anticipation of a less-complex sound environment. This might indicate that such users are aware of what the contextually most appropriate program is and proactively select it before entering a specific listening situation.

### Limitations and Future Work

This study investigates the provision and context of use of HA listening programs by analyzing data logged by HA users who also use a smartphone app. The tech-savviness and interest in listening programs of the analyzed sample should be considered when generalizing the findings from this study. In particular, older and less–tech-savvy HA users may encounter fewer complex listening environments and therefore benefit less from multiple programs [55].

In terms of future work, it would be interesting to investigate the extent to which the provision of listening programs depends on HA users requesting a program or on the hearing care professional recommending it. Indeed, hearing care professionals traditionally have a great influence on the prescribed hearing solution, and data about the provision of listening programs might not only reflect the needs and preferences of HA users but also reflect the beliefs and knowledge of the professionals. Moreover, the role of individual predictors for the provision and use of listening programs deserves further investigation. Indeed, the benefit from a personalized and contextualized solution might depend on the degree of hearing loss or additional data characterizing the individuals such as age, prior experience with HAs, auditory cognitive capabilities, or suprathreshold hearing characteristics. Finally, the significant differences found in the sound environment occurring when using specific listening programs indicate that the analyzed sound environment features (SPL, NF, and SML) are promising candidates for predicting the selection of an additional listening program over the default program. Complementing such objective sound environment features with more subjective contextual features and with an evaluation of the listening experience (eg, via an ecological momentary assessment) might also enable a deeper understanding of the provision and use of HA listening programs.

## Abbreviations

HA: hearing aid
LME: linear mixed effect
NF: noise floor
SML: sound modulation level
SPL: sound pressure level
